# Robot-Assisted Radical Cystectomy in the Management of Bladder Cancer

**DOI:** 10.1100/tsw.2006.396

**Published:** 2006-03-28

**Authors:** Jessica L. Keim, Dan Theodorescu

**Affiliations:** Paul Mellon Prostate Cancer Institute and Department of Urology, University of Virginia Health Sciences Center, Charlottesville, VA, USA

**Keywords:** bladder neoplasms, cystectomy, laparoscopy, robotics, urinary diversion

## Abstract

The application of robotic technology to laparoscopic surgery has the potential to revolutionize the entire field of urology. The use of robotic-assisted radical cystectomy has been demonstrated in the literature only within the past 3 years, as much of the reconstruction and urinary diversion techniques associated with radical cystectomy are considered more technically challenging than other procedures. Here we review the available literature pertaining to this procedure, which consists of a limited number of case reports, case series, and pilot or feasibility studies. While theses results seem to point towards less blood loss, lower transfusion rates, and shorter hospital stays compared to open radical cystectomy, definitive conclusions and recommendations cannot yet be made because of a lack of larger and/or prospective studies or randomized trials.

## INTRODUCTION

Bladder cancer has been established as the fourth most common cancer in men and the eighth most common cancer in women in the U.S.[1]. While superficial tumors can be treated effectively with transurethral resections (with or without intravesical chemotherapy), muscle-invasive bladder cancer is often treated with radical cystectomy[2]. The advent of nerve-sparing cystectomy[3] and orthotopic bladder substitution[4] has provided many patients with an enhanced quality of life, as well as excellent regional control of disease[2]. In men, the goal of a radical cystectomy is to remove the bladder with its perivesical fascia, peritoneal covering, the prostate, and seminal vesicles together with the pelvic lymph nodes[2]. In women, the bladder, uterus, adnexa, and upper half of the vagina, as well as pelvic lymph nodes, are removed in the standard operation, although some have advocated a more limited operation in premenopausal patients[5].

During open radical cystectomy (ORC), direct exposure requires a long, vertical abdominal incision from the symphysis pubis to at least the umbilicus or longer, depending on the diversion chosen[2]. This procedure can be associated with complications even when carried out by expert surgeons[2]. For example, up to 82% of women and 55% of men require a blood transfusion postoperatively as a result[6]. This present review will trace the history of laparoscopic cystectomy, as well as describe the advent of robotic-assisted laparoscopic cystectomy.

## LAPAROSCOPIC CYSTECTOMY

Laparoscopic surgery in urology started over a decade ago initially with varicocele repair, removal of pelvic lymph nodes for prostate cancer followed by nephrectomy[7]. This technology has been embraced as a result of its ability to reduce patient morbidity and maintain excellent outcomes. The first published laparoscopic radical cystectomy (LRC) for bladder cancer with an extracorporeal ileal conduit was reported by Sanchez de Badajoz et al. in 1995[8]. Most reported series in the literature were accomplished by a 5- to 6-port transperitoneal technique, and less than 10% used the hand-assisted approach[9]. LRC can be a lengthy operation (mean is 6.8 h), although this depends on the type of urinary diversion selected[9].

Basillote and colleagues reported a minor complication rate of 15% among LRC candidates vs. 45% among those who underwent the open procedure[10]. LRC is suggested to have several advantages over ORC including less fluid loss from the bowel during surgery, less blood transfusion requirements[9], lower postoperative pain analgesic needs, normal diets and bowel function are regained earlier, and superior cosmesis[11]. The laparoscopic approach was associated in some, but not all, series with reduced hospitalization. Cathelineau and colleagues observed a length of stay of 8.4 days in ORC compared to 5.1 days for LRC[12], while Balaji and colleagues suggested that the mean time to hospital discharge of 7.3 days after a LRC was not significantly shorter compared with that of contemporary ORC with a mean length of stay of 7 days[13].

The mean (range) estimated blood loss reported for LRC is 400 (245–750) ml[9]. Gill et al. reported a blood loss of more than 1 l in each of the first two reported cases of totally intracorporeal laparoscopic radical cystoprostatectomy and ileal conduit urinary diversion[14]. Meanwhile, Cathelineau reported an average blood loss of 550 ml and a transfusion rate of 5% (84 patients)[12]. In contrast, blood loss and transfusion rate was reported as 1050 ml and 25%, respectively, among patients of comparable ages and stages undergoing the open technique (70 patients)[12]. Mean transfusion rates can range from 0–30%, which progressively decrease with increasing surgical experience[9].

Early data suggest that LRC and ORC are oncologically equivalent[9]. Additionally, extended lymph node clearance is deemed possible laparoscopically in many patients[9]. Cathelineau found results that support the utility of laparoscopy for management of bladder cancer with minimal risks of tumor dissemination or trocar site implantation[12]. At an average of 18 months of follow-up for 84 patients, there were no cases of trocar site recurrences and 83% remained disease free[12]. However, this complication has been reported once with robotic laparoscopic cystectomy[15].

Some laparoscopic techniques are more difficult to perform than the corresponding tasks in open surgery due to the need for specific maneuvers such as intracorporeal suturing[16,17]. This makes radical cystectomy and urinary diversion among the most difficult procedures being performed in laparoscopic urology, especially when the urinary diversion is constructed laparoscopically[5]. The reports of LRC have included laparoscopy alone, laparoscopy with extracorporeal diversion[8,18,19,20,21], with intracorporeal ileal conduit[14], and with different forms of continent urinary diversion[22,23,24] created intra- or extracorporeally.

## USE OF ROBOTIC-ASSISTED SURGERY IN UROLOGY

Robots were first introduced into the operating room during the mid 1980s, particularly in the fields of neurosurgery and orthopedic surgery, where the anatomy provides fixed constant landmarks[25]. Advanced robotic surgery was first introduced in urology in 2000, but was slower to develop because of the challenges imposed by deformability, high mobility of urologic organs, and difficulty in reaching soft-tissue targets[25]. Most of the recent reports pertaining to the application of robotic surgery in urology have been for the management of localized cancer of the prostate (radical prostatectomy), bladder cancer (radical cystectomy and urinary diversion), kidney cancer (nephrectomy, donor nephrectomy, and pyeloplasty), and adrenal surgery[26]. The robot-assisted approach does offer some potential advantages over laparoscopy alone including increased maneuverability, improved vision due to three-dimensional images, improved dexterity due to “wristed” instruments, reduced learning curve, and by virtue of facilitating suturing, permitting reconstructive techniques that are otherwise difficult to execute using conventional laparoscopy[16,26]. Furthermore, there is reduced fatigue for the operating surgeon using robotic techniques[16].

The da Vinci system (Intuitive Surgical, Sunnyvale, CA) is currently the most sophisticated robotic instrument available for urologic procedures. It consists of two main components: a control console operated by the surgeon, and a surgical cart with three or four arms that hold a “two-eyed” laparoscope and two or three detachable tools[27]. The robotic arms are controlled by manipulating two master controls mounted on the surgeon’s console, where the surgeon sits and operates during the procedure, the head tilted forward and eyes looking through binoculars providing three-dimensional vision[27]. The surgeon then manipulates the controls that use a computerized processor that filters, scales, and relays the exact movements of the surgeon’s hands and fingers to the endoscopic instruments, thereby eliminating tremors and insignificant movements[27]. The EndoWrist® instruments offer a potential advantage over standard laparoscopy in the accuracy of suturing and the ability to dissect and grip tissues with various tools, mimicking a surgeon’s hand movement[26,27]. Additionally, da Vinci provides variable magnification of the operative field. Some of the limitations of the da Vinci system include lack of tactile feedback, cumbersome robotic installation, and high costs[16]. As of October 2005, 334 da Vinci units were in operation and of these, only ~20% were not used for urologic procedures (source: Intuitive Surgical). Furthermore, these systems were used to carry out ~10% of radical prostatectomies in the U.S. (source: Intuitive Surgical).

## ROBOTIC-ASSISTED RADICAL CYSTECTOMY

For the current review, a search was performed in PubMed and Medline from January 2002 to October 2005 with the keywords: “robotic”, “robot”, “laparoscopy” intersected with “radical cystectomy” and “radical cystoprostatectomy” within the English language. This search found six studies including case reports, feasibility studies, and case series. These are shown in Table 1.

Robot-assisted radical cystectomy and radical cystoprostatectomy are difficult robotic procedures[17]. Robotic-assisted radical cystectomy can be combined with different forms of urinary diversion (W-pouch or ileal conduit). Moreover, a neobladder can be performed *in situ*, but operative time can be reduced if this is done extracorporeally through the incision used to deliver the cystectomy[27]. The technical aspects of this procedure have been thoroughly described in several publications[5,13,17,28,29] and will not be restated here.

The first case of da Vinci–assisted cystectomy and ileal neobladder was performed by Binder and colleagues in Frankfurt, Germany in 2002 in 510 min with less than 200 cc estimated blood loss[27]. This procedure combines the oncological concepts of open surgery with the technical advantages of robotic surgery because this approach increases surgical accuracy[5]. The stable and precise movement of the EndoWrist® that allows the surgeon to dissect, divide, suture, and anastomose with great precision[5,16] may translate to reduced blood loss and morbidity[17]. In one series, most patients were often eating within 24–36 h and discharged from the hospital within 4 or 5 days[17]. Furthermore, Menon and colleagues proposed that a nerve-sparing procedure is beneficial for sexually active men whereas the minimally invasive technique in women allows for the preservation of the reproductive organs and sexual function[5,28]. The new approach is associated with a mean hospital stay of 6.7 days (range 5–8 days) among the women who had this approach[5]. Similarly, in Balaji’s pilot study demonstrating a totally intracorporeal robot-assisted laparoscopic ileal conduit urinary diversion, the mean length of stay was 7.3 days (range 5–10 days)[13].

**TABLE 1**
**Published Articles on Robotic Radical Cystectomy and Urinary Diversion**

| Published Studies, Ref. No. | No. of Cases | Ports Placed | Mean Operation Time (min) | Mean Blood Loss (cc) | Urinary Diversion | Surgical Margin | Lymphadenectomy | Complications or Conversion |
|---|---|---|---|---|---|---|---|---|
| Beecken et al.[29] | 1 Male | 5 | 510 | 200 | Hautmann ileal neobladder | Negative margins, no metastases | Bilateral pelvic lymphadenectomy (BPL) | None |
| Menon et al.[28] | 14 Male, 3 female (nerve-sparing approach) | 6 | RRCP, 140; Ileal conduit, 120; neobladder, 168 | <150 | Ileal conduit, 3; W-pouch, 10; double chimney, 2; T pouch, 2 | Negative margins, N1 disease in 1 patient | BPL; 4–27 lymph nodes removed | Not completed in 1 due to malfunction of lens; 1 port-site hematoma |
| Yohannes[30] | 2 Male | 5 | 660 | 1117.5 | Ileal conduit, 2 | Perivesical invasion, 1 case | BPL | None |
| Menon et al.[5] | 3 Females (anterior approach, 1; new technique, 2) | 6 | RRC, 160; ileal conduit, 130; neobladder, 180 | <100 | Ileal conduit, 1; W-pouch, 1; T pouch, 1 | Negative margins | BPL; 3–21 lymph nodes removed | None |
| Balaji[13] | 2 Male, 1 female | 5 | 691 | 250 | Ileal conduit, 3 (all intra-corporeally) | Negative margins | BPL | 1 Self-limiting ileus, resolved with conservative treatment |
| Hemal[17] | 21 Males, 2 females (nerve-sparing approach in males, new technique in 2 females) | 6 | RRC and RRCP, 140; urinary diversion, 150 | 200 | Ileal conduit, 4; W-pouch, 16; double chimney, 2; T-pouch, 2 | Negative margins, N1 disease in 1 patient | BPL; 3–27 lymph nodes removed | None |

The concerns about intraoperative blood loss and subsequent need for transfusion have always been associated with ORC[6]. Robotics emerged as a substantial opportunity to ameliorate the disadvantages created by laparoscopic surgery, while providing improved approaches toward nerve and reproductive organ-sparing approaches. Meanwhile, the specimen can be removed through a smaller abdominal incision or, in women, vaginal incision if intracorporeal diversion is planned. Robotic-assisted radical cystectomy is feasible and can be combined with different forms of urinary diversion (i.e., W-pouch or ileal conduit). The neobladder may be performed *in situ*, but operative time can be reduced if this is done extracorporeally through the incision used to deliver the cystectomy specimen[27]. Some authors have concluded that the ability to replicate open surgery with the same adequacy of lymph node dissection and oncologic control (avoiding tumor spillage) may still prove to be major challenges[16] in the radical cystectomy procedure. This remains to be conclusively shown in clinical studies. However, as techniques in robot-assisted cystectomy and urinary diversion improve, surgeons may witness even a decrease in blood loss and hospital stay similar to the trends noticed in robot-assisted prostatectomy. Hemal and colleagues demonstrated that none of his patients were given an intraoperative blood transfusion[17].

## CONCLUSIONS

In summary, many of the articles focusing on robotic-assisted radical cystectomy are limited to case reports and pilot or feasibility studies, performed at a single institution by a single surgical team, thereby increasing potential biases (with little follow-up and small samples). There is a critical need for long-term follow-up with cancer-free and overall survival, along with function and quality of life outcomes[17]. Presently, efficacy is poorly understood in terms of clinical applicability, patient-advantages, oncological equivalence, and cost effectiveness[16].

Until these issues are conclusively resolved, patient selection (i.e., thin patient, small stage T2 tumor) for this procedure should be stringent so as not to compromise the quality of cystectomy (i.e., positive margins). Furthermore, a thorough lymphadenectomy must be accomplished and the procedure should not deny the patient the functional benefits of a continent diversion. The latter two procedures can be carried out in an open fashion. Applying robotic technology and maneuverability to radical cystectomy has the potential to produce surgical advances, but larger and more in-depth studies are needed to determine the extent of these advantages if any.

## BIOSKETCHES

**Jessica L. Keim**, **MS**, Research coordinator, Department of Urology, Box 422, University of Virginia Health Sciences Center, Charlottesville, VA 22908. Tel: (434) 924-0042/Fax: (434) 982-3652.

**Dan Theodorescu**, Department of Urology, Box 422, University of Virginia Health Sciences Center, Charlottesville, VA 22908. Tel: (434) 924-0042/Fax: (434) 982-3652.
